# Requirements and availability of prey for northeastern pacific southern resident killer whales

**DOI:** 10.1371/journal.pone.0270523

**Published:** 2022-06-27

**Authors:** Fanny Couture, Greig Oldford, Villy Christensen, Lance Barrett-Lennard, Carl Walters

**Affiliations:** 1 Institute for the Oceans and Fisheries, University of British Columbia, Vancouver, British Columbia, Canada; 2 Ocean Wise Research Institute, Marine Mammals Research Program, Vancouver, British Columbia, Canada; 3 Fisheries and Oceans Canada, Pacific Region, Vancouver, British Columbia, Canada; 4 Raincoast Conservation Foundation, Cetacean Conservation Research Program, Sidney, British Columbia, Canada; Texas A&M University, UNITED STATES

## Abstract

The salmon-eating Southern Resident killer whale (SRKW) (*Orcinus orca*) population currently comprises only 73 individuals, and is listed as ‘endangered’ under the Species at Risk Act in Canada. Recent evidence suggests that the growth of this population may be limited by food resources, especially Chinook salmon (*Oncorhynchus tshawytscha*). We present spatio-temporal bioenergetics model for SRKW in the Salish Sea and the West Coast of Vancouver Island from 1979–2020 with the objective of evaluating how changes in the abundance, age-structure, and length-at-age of Chinook salmon populations has influenced the daily food consumption of the SRKW population. Our model showed that the SRKW population has been in energetic deficit for six of the last 40 years. Our results also suggested that the abundance of age-4 and age-5 Chinook salmon are significant predictors of energy intake for SRKW. We estimated that the annual consumption (April-October) of Chinook salmon by the whales between 1979 and 2020 ranged from 166,000 216,300. Over the past 40 years, the model estimated that the contribution in the predicted SRKW diet of Chinook salmon originating from the Columbia River has increased by about 34%, and decreased by about 15% for Chinook salmon stocks originating from Puget Sound. Overall, our study provides an overview of the requirements and availability of prey for SRKW over the last 40 years, while supporting the hypothesis that SRKW were limited by prey abundance in the study period.

## Introduction

The Northeastern Pacific resident killer whale population encompasses three genetically isolated sympatric assemblages: the Alaska residents (ARKW), the southern residents (SRKW), and the northern (NRKW) residents [1, 2]. The SRKW and NRKW are the two resident populations known to forage along the southern Coast of British Columbia during the summer months. The SRKW are often found in waters off southern Vancouver Island and Northern Washington State, while the NRKW most commonly occurs in Johnstone Strait and Queen Charlotte Strait [3, 4]. The SRKW population has received attention in recent years because of its small population size (currently 73 individuals) and recent declines. Since 2008 it has been listed as endangered in both the US [5] and Canada [6]. Important aspects of large marine mammal biology that make killer whales at risk include their slow-reproduction rate [1], late sexual maturity [7], small population size [8], and low levels of genetic variation [1]. Understanding how those inherent traits increase the vulnerability of killer whale populations worldwide remains a challenge, as the status of most of the populations is still unknown [9]. Three additional factors have been identified as potential threats for SRKW, including vessel traffic and associated underwater noise [10, 11], toxic contaminants [12, 13], and declines in food resources [14, 15]. The historical range of the SRKW population is thought to extend from California to Haida Gwaii on the north coast of British Columbia [8, 16]. SRKW historically spent about 80% of their time in the Salish Sea during the summer months, where they travel extensively through Juan de Fuca, the Haro Strait, and the southern part of San Juan Islands [17]. In recent years sightings in British Columbia are closely tied with salmon runs from May to October at the southern end of Vancouver Island [3, 18].

SRKW are highly specialized salmonids predators with a strong selectivity for Chinook salmon, which represent up to 90% of their diet during the summer months [19–21]. Chum (*Oncorhynchus keta*) and coho (*Oncorhynchus kisutch*) salmon are considered secondary prey [19, 20]. Most wild Chinook salmon populations of the northeastern Pacific have recently experienced a decline in abundance and productivity [22–24]. In Puget Sound, it was shown that although the hatchery-produced population have been relatively stable over time and have exhibited increased rates of survival and productivity, wild Chinook population have been declining since the 1970s [25]. Chinook salmon were thought to be particularly abundant in Washington and Oregon States prior to the 1990s [26]. Despite a sharp decline in the 1990s, Columbia River Chinook have shown recent signs of recovery, annual runs exceeding a million fish in some years [27]. In the Strait of Georgia and along the West Coast of Vancouver Island, most conservation units (i.e.) have seen a decrease in spawner abundance since the mid-1990s [28]. Broad scale ocean and atmospheric variation such as the North Pacific Gyre Oscillation (NPGO) and the Pacific Decadal Oscillation (PDO) are thought to be the main drivers of productivity, while changes in abundance also are linked to other factors [23, 29]. Changes in population demographic (i.e. lower size-at-age, age-at-maturity, and fecundity) [22], assimilation of pollutants [30], degradation of freshwater habitats [31], increased predation [32], and selective fisheries exploitation for larger fish [22] are recognized as other contributing factors to the decline in Chinook salmon populations along the northeastern Pacific coast [22]. For instance the high natural mortality observed in juvenile Chinook salmon in the Salish Sea might also indicate increasing predation by growing populations of harbor seals and sea lions [33–36].

Currently, half of the Chinook salmon stocks in British Columbia are in the red status zone (i.e. spawning abundance is likely to be lower than the biological benchmark needed to sustain those populations) of the Wild Salmon Policy (WSP), which was implemented in 2005 by the Canadian government to promote wild salmon conservation [37]. Most stocks spawning in the interior of British Columbia have been listed as endangered or threatened by the Committee on the Status of Endangered Wildlife in Canada (COSEWIC). In the USA, most Chinook stocks originating from the Columbia and Sacramento Rivers are currently listed as endangered or threatened under the Endangered Species Act (ESA) [38]. The decline in Chinook salmon populations has been suggested as the most important factor affecting the SRKW population demographics. Yet, evidences supporting this hypothesis are mostly limited to two periods of prey shortages, the most recent of which being around the early 2000s [39]. Additional analysis is needed to understand the magnitude of food limitation and its impact on the SRKW population. Previous research showed that food limitation for SRKW might be associated with an increase in mortality [39], decline in reproductive success [15, 40, 41], and change in social network structure [42]. Other studies suggest that the interaction between prey shortage and the SRKW population limitation remains unclear and needs further investigations [41, 43].

The energy requirements of warm-blooded, large marine mammals are particularly high due to metabolic requirements of thermoregulation and the high bioenergetics costs of foraging [44, 45]. In addition to prey abundance, there is a need to consider the prey energy value and body conditions when assessing the potential impact of food limitation on SRKW. Between 1950 and 1975, the mean weight of Chinook salmon caught in the Canadian Pacific troll fishery decreased dramatically [46]. In the southern waters of British Columbia, this decrease in mean weight was roughly 45% from approximately 4.5–5.5 kg (10–12 lbs) to 2.7–3.1 kg (6–7 lbs), apparently stabilizing in the early 1960s [46]. Since then, there have been indications that changes to fishing regulations have helped increase the mean weight of some Chinook salmon stocks, though abundances have declined for stocks in the Salish Sea [47, 48]. Some evidence suggests that the mean size of fish in southern BC stocks remains low compared to historical levels though stock-specific length and weight-at-age data is sparse. A combination of declines in Chinook salmon abundance [28, 49], reduced numbers of older (i.e. larger) fish [22, 25, 50], and potential slower growth and reduced weight-at-age [47] would threaten the SRKW population, as their high degree of prey specialization makes them particularly sensitive to changes in prey availability and population structure [39]. In this context, understanding how the consumption of fewer and smaller fish could impact the SRKW ability to meet their energetic needs is essential to promote the recovery of this population.

To address this question, we built a size and age-structured population reconstruction model of Chinook salmon stocks in the Salish Sea and along the West Coast of Vancouver Island covering the last 40 years, along with a bioenergetics model for SRKW. The ultimate objective of this paper was to evaluate potential energy deficiency patterns for SRKW over the last 40 years, while (1) understanding how variations in Chinook salmon abundance and average body size have influenced the daily food consumption of the SRKW population, (2) evaluating how potential changes in the age-structure of Chinook salmon stocks available in the SRKW range might have impacted the amount of energy available and the demographic trend of the SRKW population, and (3) examining potential variations in Chinook salmon stocks-of-origin consumed by SRKW over the years.

## Materials and method

**Fig 1:**
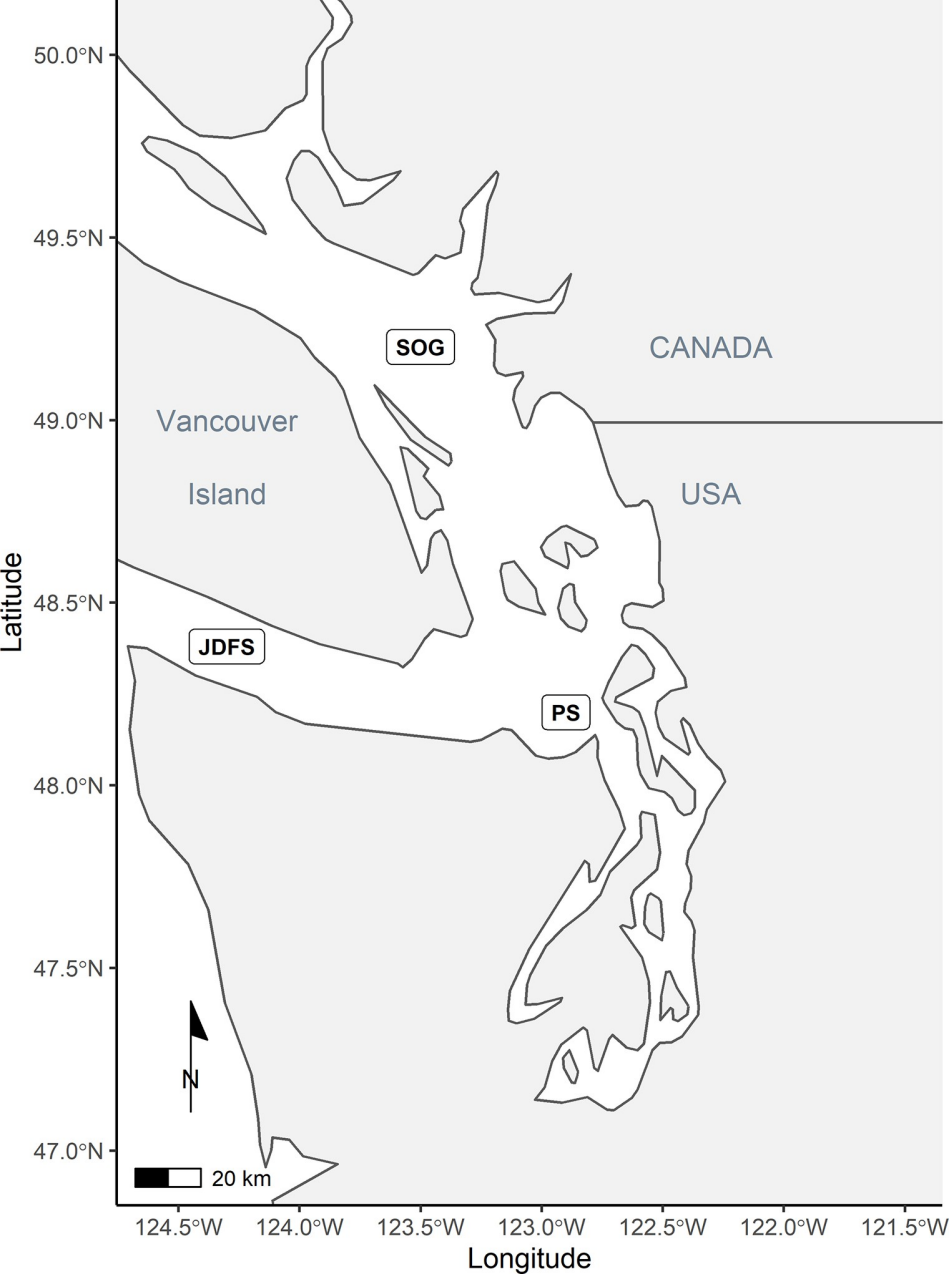
Map of the study area, including the Salish Sea (Strait of Georgia (SOG), Puget Sound (PS), and Juan da Fuca Strait (JDFS)), as well as the West Coast of Vancouver Island.

The SRKW bioenergetics model was built to represent three seasons–spring (April/May), summer (June/July), and fall (August/September/October) from 1979 to 2020. The study area for our model includes the Salish Sea (Strait of Georgia, Puget Sound, and Juan da Fuca Strait) as well as the West Coast of Vancouver Island, where SRKW chiefly are spotted between April and October [51] (Fig 1). Our model does not include winter, as it remains unclear where SRKW occur during this period.

### 1. Prey parameters estimates

#### 1.1. Chinook salmon abundance

In the early spring, SRKW are sighted most often in the western Strait of Juan de Fuca, and off SW Vancouver Island and the Olympic Peninsula, feeding primarily on spring-run Chinook salmon originating from the Columbia River and the upper portion of the Fraser watershed [20, 43, 51]. By June, SRKW spend the majority of their time in the southern portion of the Salish Sea, and are often sighted around the west coast of Vancouver Island, Juan Da Fuca Strait, San Juan Island, and Haro Strait [20]. During the summer months, Chinook salmon constitutes up to 90% of the SRKW diet, about 90% of which are summer-fall run originating from the Fraser River and travelling up the San Juan Islands between June and September [20, 44]. Adult SRKW exhibit strong prey selectivity and mainly feed on Chinook salmon aged from 3 to 5 years old, with age-4 fish being the most heavily represented in their diet (which can represent up to 50% of their Chinook salmon catches) [19, 43]. Although age-2 Chinook salmon constitute about 25% of the SRKW in the winter months, the whales appear to predominantly target older age classes of Chinook salmon in the summer months [19, 43]. For this reason, younger Chinook salmon age classes (younger than 3 years old) were not included in our model. Previous studies have used genetic methods to identify the Chinook salmon stocks targeted by SRKW between May and October [20, 43, 52]. Following those findings, we included 30 Chinook salmon stocks of interest in our model (Table 1).

**Table 1:** List of the 30 Chinook salmon stocks of interest for SRKW.

Area	Stocks	ID	Run	Area	Stocks	ID	Run
**FR**	Fraser spring 1.2	FS2	spring	**WCVI**	WCVI wild	WVN	fall
	Fraser spring 1.3	FS3	spring		WCVI hatchery	WVH	fall
	Fraser summer 1.3	FSS	summer	**OC**	North Oregon migrating	NOC	fall
	Fraser summer 0.3	FSO	summer	**WC**	Washington wild	WCN	fall
	Fraser Chilliwack fall	FCF	fall		Washington hatchery	WCH	fall
	Fraser Harrison fall	FHF	fall	**CR**	Cowlitz fall-Tulle	CWF	fall
**SoG**	Middle Georgia Strait	MGS	fall		Cowlitz spring	CWS	spring
	Lower Georgia Strait	LGS	fall		Wells Summer	SUM	summer
**PS**	Nooksack fall	NKF	fall		Mid-Columbia Brights	MCB	fall
	Nooksack spring	NKS	spring		Lyons Ferry fingerling	LYF	fall
	PS yearling	PSY	fall		Priest Rapid	URB	fall
	PS wild	PSN	fall		Bonneville Lower River	BON	fall
	PS fingerling	PSF	fall		Willamette spring	WSH	spring
	Skagit	SKG	fall		Spring Creek	SPR	fall
	Snohomish	SNO	summer				
	Stillaguamish	STL	fall				

The ‘ID’ refer to the ‘model stock acronyms’ used in the PSC model, and in this paper. ‘Run’ refers to the seasonal timing of the spawning migration for each stock. ‘Stocks’ are shown by ‘Area’ of origin, which include the Fraser River (FR), the Strait of Georgia (SoG), Puget Sound (PS), the West Coast of Vancouver Island (WCVI), Oregon Coast (OC), Washington Coast (WC), and the Columbia River (CR).

The method described below in our reconstruction model yield ranges of annualized Chinook salmon numbers-at-age estimates for mature or fish nearing maturity (at least 2 years of ocean age, Ocean Age 2+). In total, 30 stocks were included given that their migratory route out of the Salish Sea as juveniles and back as adults requires them to pass through at least the southwestern portion of the study area (i.e. Southwestern part of Vancouver Island, Juan de Fuca Strait).

The PSC is an international organization formed to support implementation of the Pacific Salmon Treaty between Canada and the United States. The Chinook Technical Committee (CTC) of the PSC publishes annual public reports on Chinook salmon catch and escapement, Coded-wire-tag (CWT) exploitation rate analysis, and PSC Chinook Model calibrations. An advantage of the CTC Model is the large breadth of integrated data. The PSC expends a large effort to integrate coded-wire-tag (CWT) release and recovery data as well as catches from Canadian and American fisheries both inside and outside the Salish Sea for each stock and age class. The modeling method used in the CTC model is a combination of backward and forward cohort analysis, which relies on various statistics, including the base period CWT data, Canadian and American fisheries catch data, Chinook salmon non-retention data, estimates of fisheries and gear-specific catchability, escapement and non-terminal run data, maturation rate and adult equivalent data, hatchery releases and recoveries, spawner-recruit parameters, and proportion non-vulnerable estimates) [53]. Age 2+ fish (Gilbert and Rich format) have a much larger ocean distribution than the study area, with a notable proportion of fish from the Salish Sea being caught in northern Alaskan fisheries [53–56]. Modeled estimates of total cohort, escapement, catch by fishery-at-age were directly provided by the Pacific Salmon Commission (PSC) Chinook Technical Committee (CTC) for our 30 stocks of interest [53]. It should be noted that no uncertainties estimates were associated with those data.

Summing the PSC’s estimates of total escapements and catch from fisheries occurring within the Salish Sea yields a minimum abundance-at-age for each stock *s* at the start of each year within the study area, using:

$$N_{s,a,y,A}=C_{s,a,y,A}+E_{s,a,y}$$ (1)

where *N*_*s*,*a*,*y*,*A*_ is the minimum abundance of fish originating from stock *s* and age *a* transiting through the study area *A* during year *y*. *C*_*s*,*a*,*y*,*A*_ is the total catch of fish at age *a* originating from stock *s* from all fisheries operating in the area *A*. *E*_*s*,*a*,*y*_ is the escapement estimate for age *a* fish originating from stock *s* in year *y*. Assuming accurate catch reporting and escapement estimates, *N*_*s*,*a*,*y*,*A*_ represents a minimum bound on the number of fish in each age class passing through our study area each year.

Predation mortality of mature and immature fish in each cohort also undoubtedly occurs in the study area. We used the static estimates of age-specific natural mortality rates from the CTC model to expand the minimum estimate of mature fish abundance and in transit to their spawning grounds within the study area [53]. The assumed natural instantaneous mortalities were 30% for age-3 fish (*m* = 0.36 year^-1^), 20% for age-4 fish (*m* = 0.22 year^-1^), and 10% for age-5 fish (*m* = 0.1 year^-1^). As these PSC natural mortality estimates already included predation mortality, we estimated the Chinook consumption by SRKW for the different stocks from 1975 to 2020. Most consumption estimates were lower than 10% and were then considered small enough not to substantially impact the initial stock abundance calculations.

Because of a lack of information allowing us to divide catch between mature and immature fish, all catch within the study area was assumed to be of mature fish returning to their spawning grounds. Immature fish were assumed to experience predation mortality over the full year (*m*_*a*_) and escapees and fish caught in fisheries were assumed to experience predation mortality over two months prior to spawning (*m*_*a*_/6). The equation used to expand catch and escapement in the study area was:

$$N_{m,s,a,y,A}=\frac{C_{s,a,y,A}+E_{s,a,y}}{e^{-ma/6}}$$ (2)

where *N*_*m*,*s*,*a*,*y*,*A*_ is the abundance of mature fish *N*_*m*_ originating from stock *s* and age *a* transiting through the study area *A* during year *y* (prior to fishing and predation mortality), and where *m* is the natural instantaneous mortality-at-age. This method implicitly assumes that natural mortality occurs prior to fisheries mortality which was deemed acceptable for our purposes given that terminal run fisheries mortalities are generally higher than ocean fisheries mortality on Fraser River stocks. Another advantage of back-calculation is it allowed us to include only those fisheries catches that occurred in the study.

Only a portion of a cohort spawns each year (estimated in the CTC model using maturation schedules, but these were unavailable at the time of writing). In lieu, the CTC model provides an estimate of total cohort size, *N*_*s*,*a*,*y*_ which was used here to back-calculate the number of immature fish *N*_*i*_ at age *a* originating from stock *s* in year *y* as:

$$N_{i,s,a,y}=N_{s,a,y}-N_{m,s,a,y}$$ (3)

However, the CTC model estimates of abundance-at-age, *N*_*a*_, (i.e. a ‘cohort’) were sometimes *lower* than the catches for that age class in the same year, *C*_*a*_−i.e. for some years, the values calculated by subtracting age-specific catches from age-specific abundance were negative. In this situation, the cohort size estimates provided by the PSC were used instead. This abnormality might be explained by an unusual method for calculation of the total cohort size used in the CTC model. To calculate abundance of an age class where fisheries occur early in the year, a common method is:

$$N_{a+1}=s(N_{a}-C_{a}-S_{a})$$ (4)

where the abundance in the next age class, *N*_*a*+1_, is the abundance from the previous year multiplied by survival rate, *s* (i.e. *s = 1-*M where M is the finite mortality rate) minus the catches, *C*_*a*_, and spawners, *S*_*a*_, from the previous year. Thus, the survival rate is applied only to the fish remaining after harvest and excluding the fish that spawned. Using the same equation as above, the equation used in the CTC model can be rewritten as:

$$N_{a+1}=sN_{a}-C_{a}-S_{a}$$ (5)

The survival calculation in the CTC model is therefore applied to the cohort *prior* to all fishing and spawning, not after. We expressed concern to PSC on this matter. The data provided did not include the base year catch and survival estimates, and other parameters, so it was not possible to re-do the cohort reconstruction. As such, the CTC total cohort size was assumed to be biased low.

Spawning fish are not present for the entire year, so spawn timing data and spatial distribution information was used in calculation of numbers-at-age for each season in the Salish Sea and along the West Coast of Vancouver Island. The marine distribution of maturing Chinook salmon is believed to be significantly greater than the Salish Sea and the west coast of Vancouver Island, and generally thought to vary by population [56]. We found no evidence that a significant proportion of Chinook salmon originating from the Columbia River basin and the outer coast rear in the Salish Sea, so the relative proportion of immature Chinook salmon for those stocks was not accounted for. Shelton et al. (2019) investigated marine distributions of fall-run Chinook salmon on a seasonal basis, and concluded that fall-run Chinook salmon originating from the Fraser River and Puget Sound spend more time rearing in the Salish Sea than the spring- and summer-run populations [55]. For spring and summer run immature Chinook, we estimated that 5% of each cohort was present in the study area in all months based on an estimate that the study area represents approximately 5% of the marine range of the species, combined with stock-specific marine distributions summarized in Brown et al. 2019 [56]. We relied on the analysis of Shelton et al. (2019) for estimates of seasonal proportions of Fraser- and Puget Sound- origin fall-run immature Chinook salmon present within the Salish Sea and applied these estimates to arrive at a proportion of all immature fish *N*_*i*,*s*,*a*,*y*,*A*_ at age *a* originating from stock *s* and present in the study area *A* in year *y* (Table 2):

$$N_{i,s,a,y,A}=N_{i,s,a,y}\times P_{i,s,a,y,A}$$ (6)

where *P*_*i*,*s*,*a*,*y*,*A*_ is the proportion of immature fish *N*_*i*,*s*,*a*,*y*,*A*_ present in the study area *A*. Given that the total cohort size is estimated in the CTC model with natural mortality accounted for, no expansion for predation mortality was applied.

**Table 2:** Estimated seasonal proportions *P*_*i*,*s*,*a*,*y*,*A*_ of the fall and spring and summer runs immature Chinook salmon residing in the Salish Sea.

Stocks	Run	Spring	Summer	Fall
FR	spring	0.05	0.05	0.05
FR	summer	0.05	0.05	0.05
FR	fall	0.35	0.35	0.4
PS	spring	0.05	0.05	0.05
PS	summer	0.05	0.05	0.05
PS	fall	0.05	0.05	0.05
SOG	fall	0.35	0.35	0.4

The stocks are originating from 3 main areas: Fraser River (FR), Puget Sound (PS), and the Strait of Georgia (SOG).

To estimate seasonal presence for spawning fish, we used peak run timing for Fraser and Strait of Georgia stocks [57, 58] and Puget Sound stocks [59] and subtracted two weeks to account for travel time through the Salish Sea. We used these estimates as a multiplier to arrive at the proportional seasonal abundance *P*_*s*_ of mature fish originating from those stocks and travelling to their spawning grounds (Table 3). For all Chinook salmon originating from the Columbia River Basin and the Oregon/Washington Coast, we relied on 2 area-specific indices (Salish Sea (Salish) and Southwest Vancouver Island (SWVI)) provided by NOAA [60]. It is important to mention that those indices were calculated from the Chinook FRAM model, which has slightly different time-steps than our model (one-month difference). It is also important to consider that we used those estimates as a proxy for spring- and summer-run, as most of those area-specific indices were originally computed for fall-run Chinook stocks [60].

**Table 3:** Estimated seasonal proportions *P*_*s*_ of the fall run and migrating spring and summer mature Chinook salmon present in the Salish Sea and along the West Coast of Vancouver Island in the spring, summer, and fall.

Area	Stocks	spring	summer	fall	Area	Stocks	spring	summer	fall
Fraser	FS2/FS3	0.8	0.6	0	PS	SNO	0.6	0.8	0.3
Fraser	FSO/FSS	0.6	0.7	0.3	WCVI	WVH/WVN	0.055	0.07	0.24
Fraser	FCF/FHF	0	0.3	0.7	OC	NOC	0.09	0.075	0.07
SoG	MGS/LGS	0	0.3	0.7	WC	WCN/WCH	0.14	0.07	0.15
PS	NKF/SKG/STL	0.51	0.5	0.47	Lower CR	CWF/BON/SPR	0.21	0.19	0.1
PS	PSY	0.09	0.075	0.07	Middle CR	SUM/MCB/LYF	0.18	0.14	0.11
PS	PSN	0.2	0.12	0.17	Upper CR	URB	0.2	0.18	0.1
PS	PSF	0.17	0.17	0.17	CR spring	CWS/WSH	0.17	0.17	0.17
PS	NKS	0.8	0.6	0					

Fall-run Chinook salmon abundance (all stocks) and spring and summer-run abundance estimates of southern stocks (i.e. WVN, WVH, NOC, WCN, WCH, BON, CWF, SPR, SUM, MCB, LYF, URB, CWS, WSH) are based on area-specific indices provided by NOAA and Shelton et al. (2019), whereas spring and summer Chinook salmon abundance estimates for stocks originating from the Fraser River, Puget Sound, and the Strait of Georgia (i.e. FS2, FS3, FSO, FSS, NKS, SNO) are computed with Scenario 2.

Using the relative proportion of each stock present in the Salish Sea and along the West Coast of Vancouver, we estimated the relative contribution of each stock in the predicted SRKW diet between 1979 and 2020, assuming that the whales did not exhibit any preference for specific stocks. We found no studies estimating the relative proportion of spring and summer run mature Chinook salmon found within the Salish Sea and West Coast of Vancouver Island from April to October. For these populations, we ran our models under three different scenarios of seasonal distribution of those stocks in the study area (Table 4).

**Table 4:** Relative seasonal proportions *P*_*s*_ of migrating spring and summer Chinook salmon stocks originating from the Fraser River, Puget Sound, and the Strait of Georgia.

Run	Spring run Chinook salmon			Summer run Chinook salmon		
**Season**	Spring	Summer	Fall	Spring	Summer	Fall
**Scenario 1**	0.8	0.6	0	0.6	0.8	0.2
**Scenario 2**	0.6	0.5	0	0.5	0.7	0.3
**Scenario 3**	0.5	0.3	0	0.3	0.5	0.4

The relative seasonal proportion of the stocks (i.e. FS2, FS3, FSO, FSS, NKS, and SNO) was computed under three different scenarios. The results of this paper are presented for Scenario 2, as we found little sensitivity of our model to variations in abundance estimates for those different scenarios. The values are expressed as relative proportion of the stocks. Note that they do not add up to 1 as some fish might stay in the area for several weeks and be available to SRKW over several seasons.

#### 1.2. Chinook salmon length-at-age and energetic value estimates

Length-at-age models for Fraser River Chinook salmon stocks from 1979 to 2020 provided by the PSC were used in our model [61]. The mean Von Bertalanffy growth parameters from this statistical model were used here.

Chinook salmon have high energy densities (average of 1,724kcal.kg^-1^), but exhibit important variations in body sizes and energy content among Northeastern Pacific populations [62]. Yet, there is a paucity of information regarding variation of mass and lipid content in salmon populations. In this context, the linear regression model developed by O’Neill (2014) to predict the relationship between fish length (in mm) and total energy content (kcal.fish^-1^) for Chinook salmon was used, such that:

$$Kcal.fish^{–1}=0.000011{\left(fish\;fork\;length\right)}^{3.122}$$ (7)

Mass-at-age was calculated using length-weight equations for Chinook salmon from an empirical study of lab and pen-raised Strait of Georgia Chinook [63].

#### 1.3. Chum and coho salmon

In late September and October, SRKW also prey on large Fraser runs of coho and chum salmon [43]. The Fraser River is home to the largest run of chum salmon in British Columbia [64]. Fraser River chum salmon all migrate from September to December through Johnstone Strait, Juan da Fuca Strait, and the Strait of Georgia to reach their spawning grounds in the lower portion of the Fraser River [64]. In fact, coho and chum salmon may constitute between 20% and 50% of the SRKW diet during this period, and previous studies suggest that age-3 coho salmon and age-4 chum salmon are predominantly targeted [19, 43].

In the fall and early winter, coho salmon are migrating in high number to their spawning grounds in the interior Fraser River watershed [65]. Estimates of numbers of returning adult coho salmon in the Salish Sea and through the Juan da Fuca Strait were calculated using escapement numbers, historical catches, and CWT survival rates for the Strait of Georgia and the Fraser River. Escapement data were extracted from the publicly accessible database NuSEDS (i.e. New Salmon Escapement Database System) for streams in the Strait of Georgia and the Fraser River region, and expanded by the proportion of streams surveyed each year [66]. The escapement numbers were added to the historical catches reported in the public Pacific Fisheries Catch Statistic Database, and fitted to the total survival rates which were obtained from a coastwide database of tag releases and recoveries maintained by the Pacific Salmon Commission [67–69]. To obtain pre-harvest coho abundances, the escapement estimates were expanded by ratios of catch to escapement based on coded wire tagging recovery data. Coho salmon catch:escapement ratio estimates have varied from year to year depending on tag expansion assumptions, but have generally been consistent in terms of overall exploitation rates, especially after the major decrease that occurred in 1997 when retention of coho was largely stopped as a conservation measure [67]. It is important to note that some of those databases contain estimates of highly variable quality, and that some information was missing for some streams in recent years. For chum salmon, we used estimates of catch and escapement provided by the Fraser and Interior Area Stock Assessment Program and computed by the Department for the Fisheries and Oceans (corrected for changes in sampling effort) (Mike Hawkshaw, personal communication). The catch data was compiled by various catch programs, primarily validated landings at processing plant (Mike Hawkshaw, personal communication).

### 2. Predator parameter estimates

**Fig 2:**
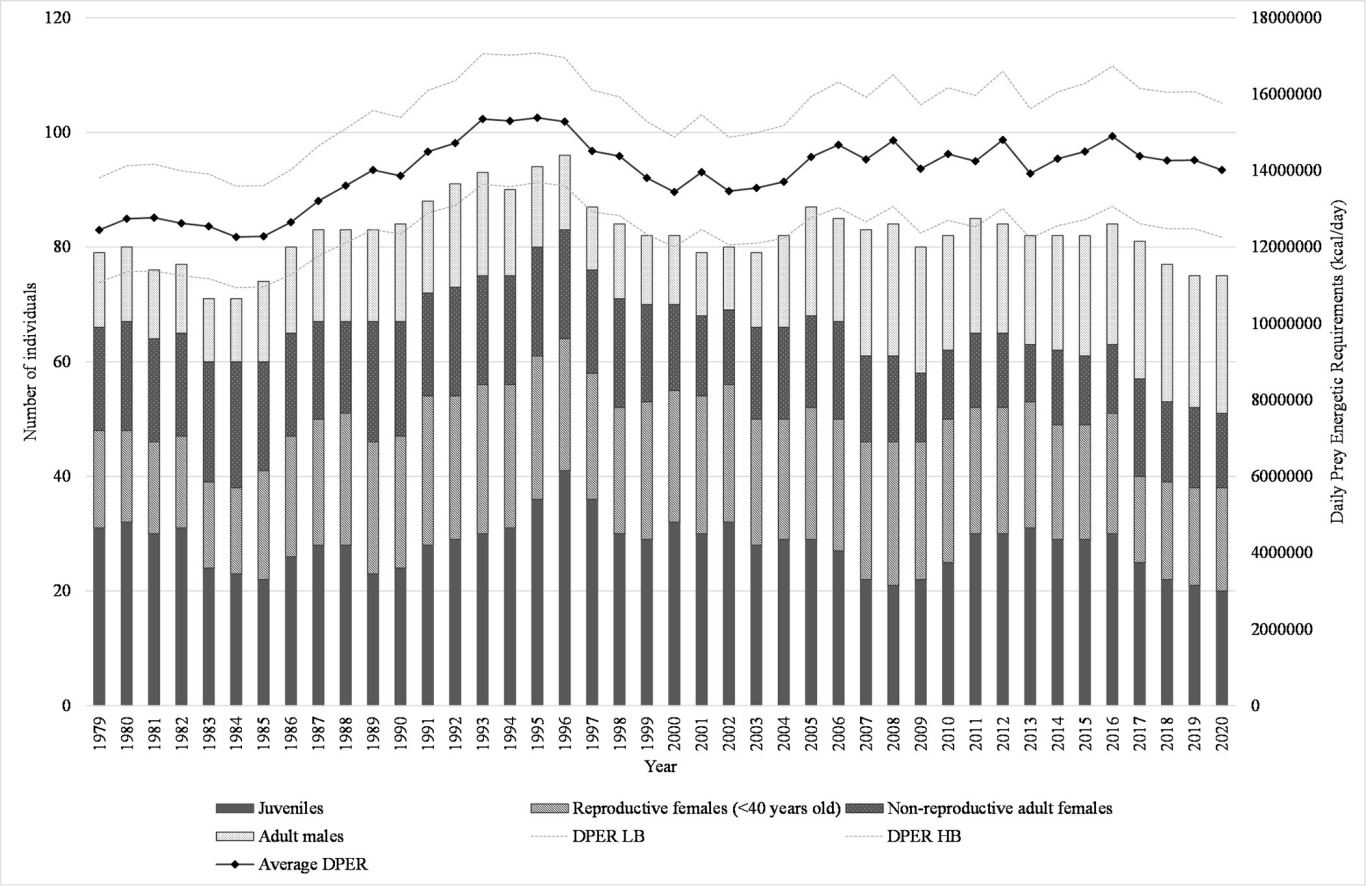
**Number of individuals (stacked columns) and associated average daily prey energetic requirements (solid line) for the SKRW population from 1979 to 2020.** The dashed lines represent the higher and lower bounds of the DPER estimates.

As energetic requirements for SRKW are impossible to accurately measure in the field, we used the Daily Prey Energetic Requirement (DPER) estimates for SRKW developed by Noren in 2011 [45]. Those DPER estimates are based on field observations of different activity states for SRKW, and account for a digestive efficiency of 84.7% [70]. As energetic needs are proportional to individual body mass, we used births and deaths data collected by the Center for Whale Research to estimate the age- and sex- structure of the SRKW population from 1979 to 2020 [71] (Fig 2). Calves younger than 1 year old were not included in our analysis, as they do not feed on solid food during the first year of their life [71].

For each year, we estimated the DPER of every whale individual based upon previous estimations of mass-at-age [45]. Following the method developed by Noren in 2011 [45], we assumed that the body mass of SRKW between 1 and 12 years of age was similar for males and females, and increased at a constant rate of 183.4 kg.year^-1^ (to reach a body mass of 2298.6 kg at 12 years of age). It was assumed that both males and females level their body growth and food consumption by 20 years of age to reach terminal body masses of 3,338kg and 4,434kg, respectively [45]. Between 12 and 20 years of age, the body mass of females was assumed to increase at a constant rate of 107 kg.year^-1^, while males grew at a constant rate of 244 kg.year^-1^. For each year, lower and upper bound DPER estimates were calculated for every whale individual using Noren’s equations [45]:

$$(a)\;\mathrm{Low}\;\mathrm{bound}\;\mathrm{DPER}:\;{413.2*M}^{0.75}\;(b)\;\mathrm{High}\;\mathrm{bound}\;\mathrm{DPER}:\;495.9*M^{0.75}$$ (8)

Where M is whale body mass (in kg), and DPER in expressed in kcal/day.

### 3. Prey-predator dynamics: The Holling disc equation

The multi-species disc equation developed by Holling predicts a type II functional response, where the prey consumption rate of a predator rises as prey density increases before levelling off as predators become limited by their capacity to process food (handling time). This equation was used in our model to predict the relationship between the SRKW rate of food consumption and the relative density of the different age classes of Chinook, chum, and coho salmon. This model allowed us to estimate the average daily prey intake by SRKW, as well as the annual consumption of Chinook salmon by SRKW in the Salish Sea and along the West Coast of Vancouver Island between 1979 and 2020. Although it is known that SRKW share their prey (especially adult females), we were able to treat each whale individual as a single predator without changing the prey handling time [19].

The Holling disc equation is routinely used to predict multispecies diet patterns in ecosystem modelling. According to the Holling disc equation, the total number of preys eaten per predator daily *P*_*t*_ can be predicted as:

$$P_{t}=\sum_{i=5}^{i}P_{i}=\sum_{i=5}^{i}((a_{i}N_{i}T_{t})/(1+\sum a_{i}\;N_{i}h_{i})$$ (9)

where for each prey *i*, *P*_*i*_ is the total number eaten per day, *N*_*i*_ the estimated abundance available to predators, *a*_*i*_ the rate of effective search, and *h*_*i*_ the handling time per prey.

To use the Holling disc equation, we first needed to calculate the Holling disc equation parameters values needed to predict the observed diet composition in some reference years for which we have estimates of total prey abundance *N*_*i*_ by prey type and relative diet proportions of those prey types in the predators’ diet. We used Ford and Ellis (2006) estimates of all prey types *i* in the SRKW diet during those reference years (here 2000–2004) to calculate the disc equation parameter values (i.e. rate of search efficiency *a*_*i*_ and handling time *h*_*i*_) needed to predict the observed diet composition during those years (Table 5). To do so, we assumed that *T*_*t*,_ which represents the total time where SRWK were ‘reactive’ to prey, was equal to 21 hours/day [45]. *T*_*t*_ can be written as:

$$T_{t}=T_{s}+\sum h_{i}\;P_{i}$$ (10)

where *T*_*s*_ was defined as the time actively searching for prey, and equal to 5 hours/day [45]. We assumed that the handling time *h*_*i*_ of each prey type *i* was proportional to prey weight *W*_*i*_, so that:

$$h_{i}=k_{i}W_{i}$$ (11)

**Table 5:** Base parameters used to calibrate the Holling disc equation.

	Age 3	Age 4	Age 5	Chum	Coho
Diet proportion *(in biomass)*	0.101891	0.424545	0.297182	0.070909	0.08
Prey abundance ***N*** *(in numbers)*	551903	309695	41916	321030	2455706
Mean length *(in cm)*	769.3913	841.0689	882.7972	700	650
Weight *(in kg)*	6.777113	8.098663	8.922202	4.3	3.5
Average energy *(in kcal*.*fish*^*-1*^*)*	11270.53	14883.9	17312.93	8390.518	6657.454
Age proportion	1.240471	5.168629	3.61804	0.863283	0.97396
Handling time ***h*** *(in hours)*	1.209285	1.445099	1.592048	0.767278	0.624528
Rate of effective search ***a*** *(in prey abundance/hours)*	4.50E-07	3.34E-06	1.73E-05	5.38E-07	7.93E-08

Under this assumption and by converting the previous equations, the weight coefficient *k*_*i*_ was calculated to estimate the handling time *h*_*i*_ per prey type *i* so that:

$$k_{i}=T_{h}/\sum P_{i}\;W_{i}$$ (12)

Where *T*_*h*_ represents the total prey handling time, which can also be written as:

$$T_{h}=T_{t}-T_{s}$$ (13)

The predator rate of search efficiency *a* can be defined as the area searched by a predator per unit of time. The kill rate of a predator depends on the searching efficiency, which itself logically varies with prey density *N*_*i*_. The predator rate of search efficiency can thus be expressed as:

$$a_{i}=P_{i}/N_{i}T_{s}$$ (14)

Similarly to the other base parameters of the Holling disc equation, the rate of search efficiency *a*_*i*_ was calculated using the number of prey type *P*_*i*_ eaten between 2000 and 2004 as well as their overall abundance estimates *N*_*i*_ over those reference years [19]. It is impossible to estimate the sensitivity to variations in the Holling disc equation parameters other than to provide results for a range of reasonable increment in critical parameters. In the case of the Holling disc equation, it is expected that the model would likely show small sensitivity to any parameters except *T*_*s*_, as this base parameter is used to calculate estimates of the rate of search efficiency *a*_*i*_ from the diet data. To address this uncertainty, we ran our model under two scenarios of *T*_*s*_ = 3h and *T*_*s*_ = 7h (±20%), and provided an estimate of the annual Chinook salmon consumption by SRKW and seasonal DPER differential for each scenario.

There is a paucity of information on the average daily food weight consumption for wild resident killer whales. The average daily energetic requirements of our SRKW population between 2000 and 2004 were about 171.245 kcal.day^-1^, and the average prey energy content was 13.740kcal (estimated by multiplying each relative prey proportion in the SRKW diet by their average energy content). Based on those estimates, we assumed that a SRKW individual consumed about 12 fish.day^-1^, or about 102 kg.day^-1^. Those estimates are about 54% higher than previous calculations on captive killer whales, which could logically reflects higher energy expenditure associated with swimming, hunting, and socializing in the wild [45, 72]. Based on our calibration for the reference years, we were able to predict the consumption of each prey *i* by SRKW in the spring, summer, and fall between 1979 and 2020. Logically, the overall daily energy *E*_*t*_ gained by the SRKW population was defined as:

$$E_{t}=(\sum_{i=5}^{i}P_{i}E_{i})N_{p}$$ (15)

Where *E*_*i*_ is the energy content of each prey *i* and *N*_*p*_ the total number of predators.

It is important to note that the big uncertainty in these calculations is the base time *T*_*s*_ spent searching for prey each day, which might vary with factors like diurnal behavioral changes and obligatory time spent socializing. It is difficult to be more precise because even if the whales were followed closely over time, it would be hard to determine whether they would be reactive to prey at any given moment should they encounter one during other activities, like socializing or “resting”. Although hard to quantify, this limitation could cause substantial uncertainty in the *a*_*i*_ ratios, along with uncertainty about diet proportions of prey of different sizes/ages/stocks.

To determine how the SRKW population was able to meet its energetic needs over the last 40 years, we compared the energetic differential between the DPER (low, high, average) and the average energy ingested per individual. We also used a logistic regression to understand which factors (i.e. abundance-at-age, length-at-age) could best predict the likelihood of the SRKW population meeting its energetic needs from April through October each year assuming the prey density dependence functional response relationship described below. In order to understand whether food limitation could influence the demographics of the SRKW population, we compared the birth rate (i.e. number of births/number of reproductive females), death rate (i.e. number of deaths/number of individuals in the population), as well as the annual net population change (birth rate–death rate) between years where the model predicted that the SRKW population met its DPER and years where it did not. We used graphical representations and a Wilcoxon rank-sum test to evaluate the presence of an energetic consumption influence on the SRKW population demographics. Our entire model was built on R (R Core Team 2013, version 1.4.1103).

## Results

Overall, the model sensitivity to changes in spring and summer stocks’ spatio-temporal distribution was considered small enough not to affect the results significantly. Between scenarios 1 and 3 (see Table 4), the overall trend of variations in average daily prey energetic requirement differentials remained comparable, with a maximum difference of 4143 kcal, or about 2.4% of the average DPER for a SRKW. Similarly, the difference between the annual Chinook salmon consumption by SRKW under those two scenarios was about 5,817 fish, which only represents about 2.5% of the total annual consumption of Chinook salmon by SRKW. No major difference in relative stock contribution into the predicted SRKW diet was found when running the model under the three different scenarios. We estimated that the overall predictions of our model did not significantly differ under our three scenarios, and chose to present the results of our model under scenario 2.

**Fig 3:**
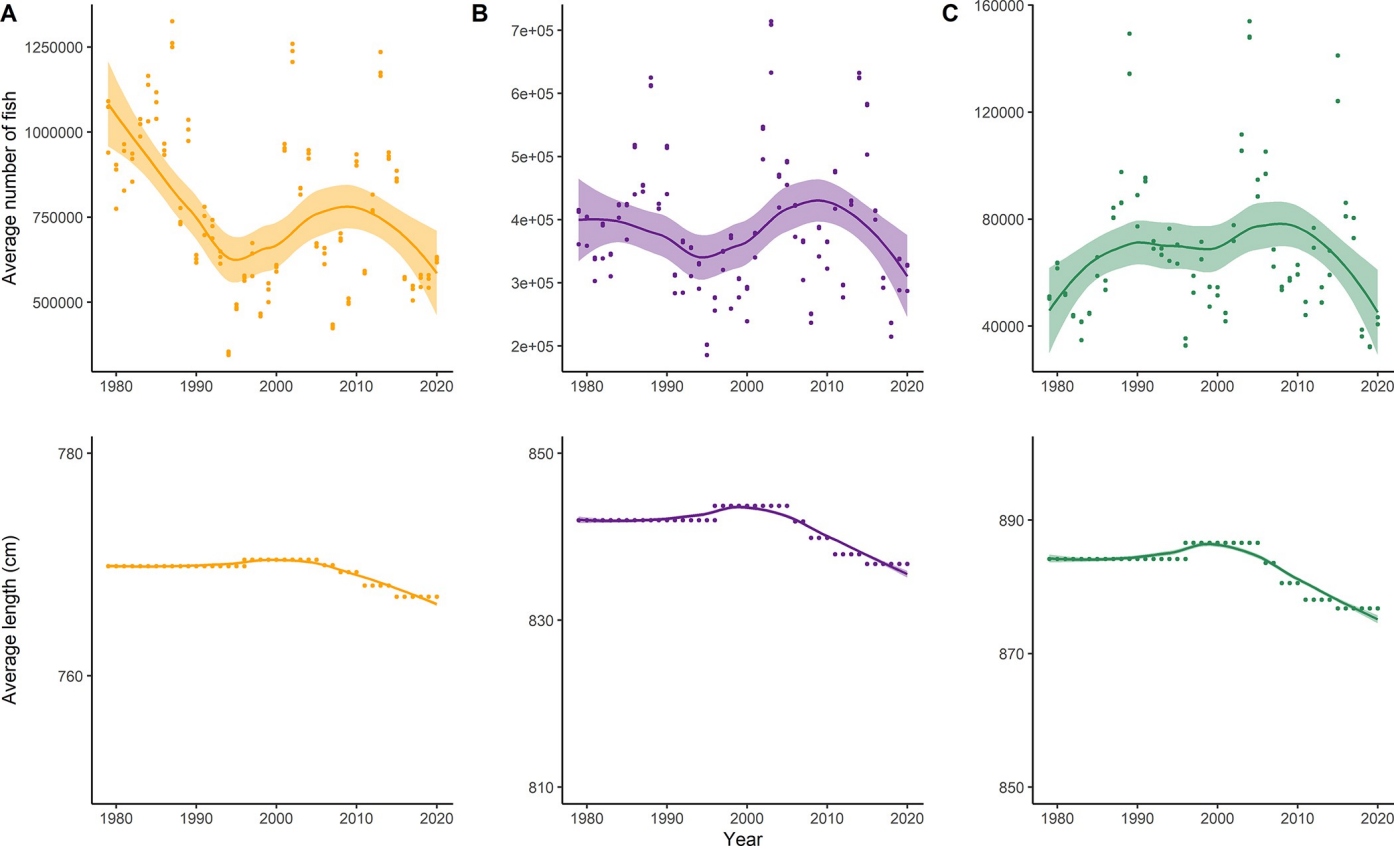
Average trend of abundance (top) and length-at-age (bottom) for age-3 (A), age-4 (B), and age-5 (C) Chinook salmon available to SRKW from 1979 to 2020 during the months April-October.

The data provided confirmed that the overall abundance of all Chinook age classes available to SRKW decreased over the study period (Fig 3). When comparing abundance-at-age numbers between 1979 and 2020, we found that the overall age-3, age-4, and age-5 fish abundance decreased by 39.7%, 20.9%, and 25.8%, respectively. The data also showed that the average abundance-at-age was the lowest during the mid-1990s, before increasing to higher levels around 2010 (Fig 3). The average length-at-age also slightly decreased for all ages of Chinook salmon targeted by SRKW, especially since 2000, by about 3cm (age-3), 7cm (age-4), and 10cm (age-5). For an age-5 Chinook salmon, a 10cm decrease in fish size would represent a difference of about 220kcal per fish.

Altogether, our model estimated that the uncertainty of our model associated with reasonable variations in the time during which SRKW were actively searching (i.e. *T*_*s*_) was relatively minimal. When running the model under *T*_*s*_ = 5±20%, we found that the average difference in the annual Chinook consumption was about 4748 fish, or ~22 fish per day for the whole SRKW population.

**Fig 4:**
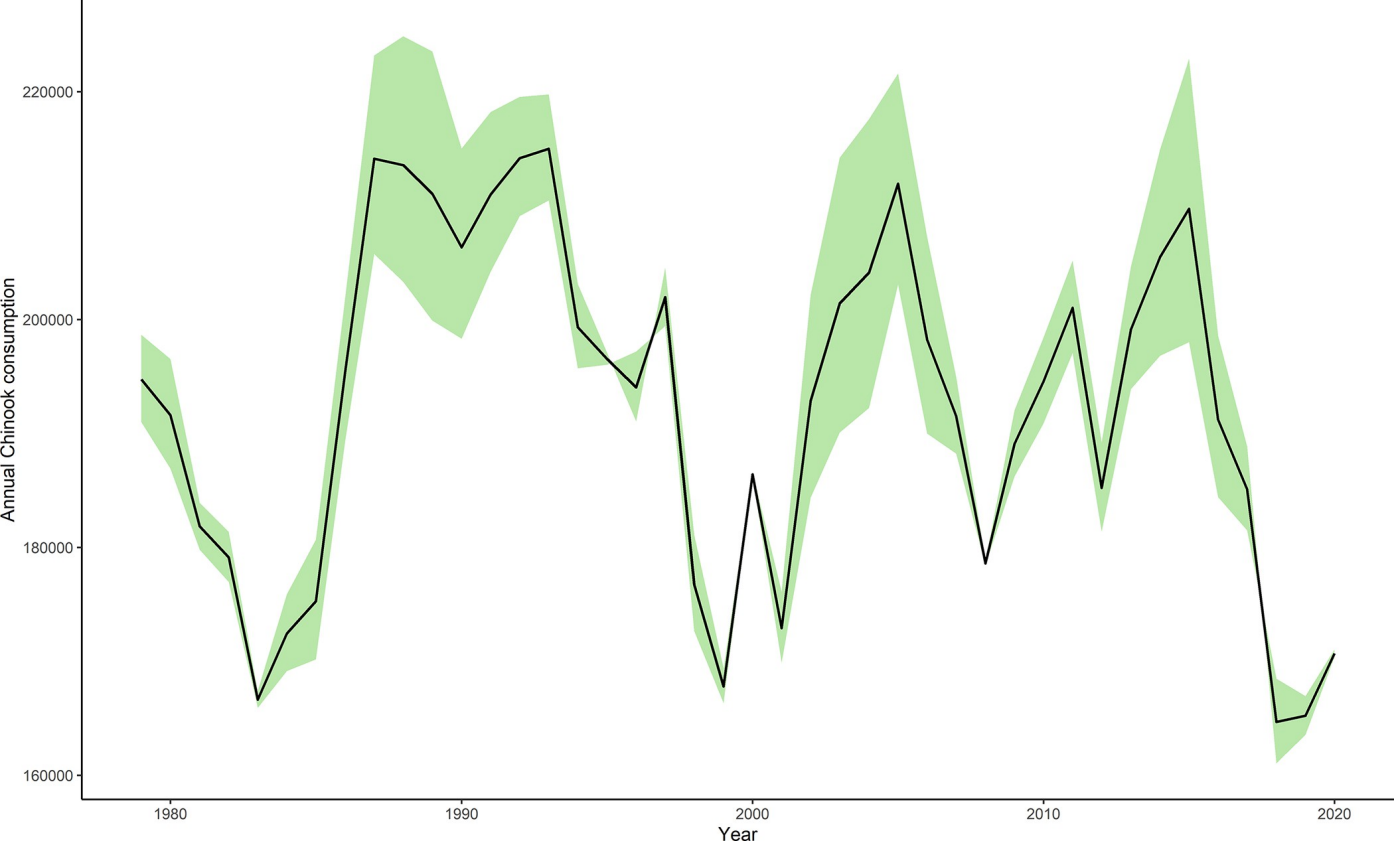
Estimated annual (excluding November-March) consumption of Chinook salmon (in number of fish) from April to October in the SRKW range from 1979 to 2020. The black line represents the predicted annual Chinook salmon consumption for *T*_*s*_ = 5h, whereas the green area represents the estimated Chinook salmon consumption for *T*_*s*_ = 5h±20%.

Using our base value of *T*_*s*_ = 5h, we estimated that the seasonal Chinook salmon consumption by SRKW from April to October varied between about 216,300 and 166,000 fish.yr^-1^ between 1979 and 2020 (Fig 4). It is worth highlighting that the trend observed regarding the annual Chinook salmon consumption by SRKW reflects temporal variations in Chinook salmon abundance as well as changes in the size of the SRKW population. The estimated highest consumption was in 1993, whereas the estimated lowest consumption was in 2018. The model predicts that consumption by SRKW of Chinook salmon belonging to the stocks in the model dropped substantially twice over the study period. A 22% decrease in Chinook salmon consumption was observed between 1993 and 1999, whereas consumption dropped by 18.4% between 2015 and 2020. Our model showed that chum salmon consumption by SRKW oscillated between 53,887 and 4,710 fish per year (highest level in 1998 and 1999), while coho consumption varied between 6,115 and 382 fish only. Our model predicted that SRKW consumed more chum salmon in the fall than age-3, age-4, and age-5 Chinook salmon separately in 1998 and 1999.

**Fig 5:**
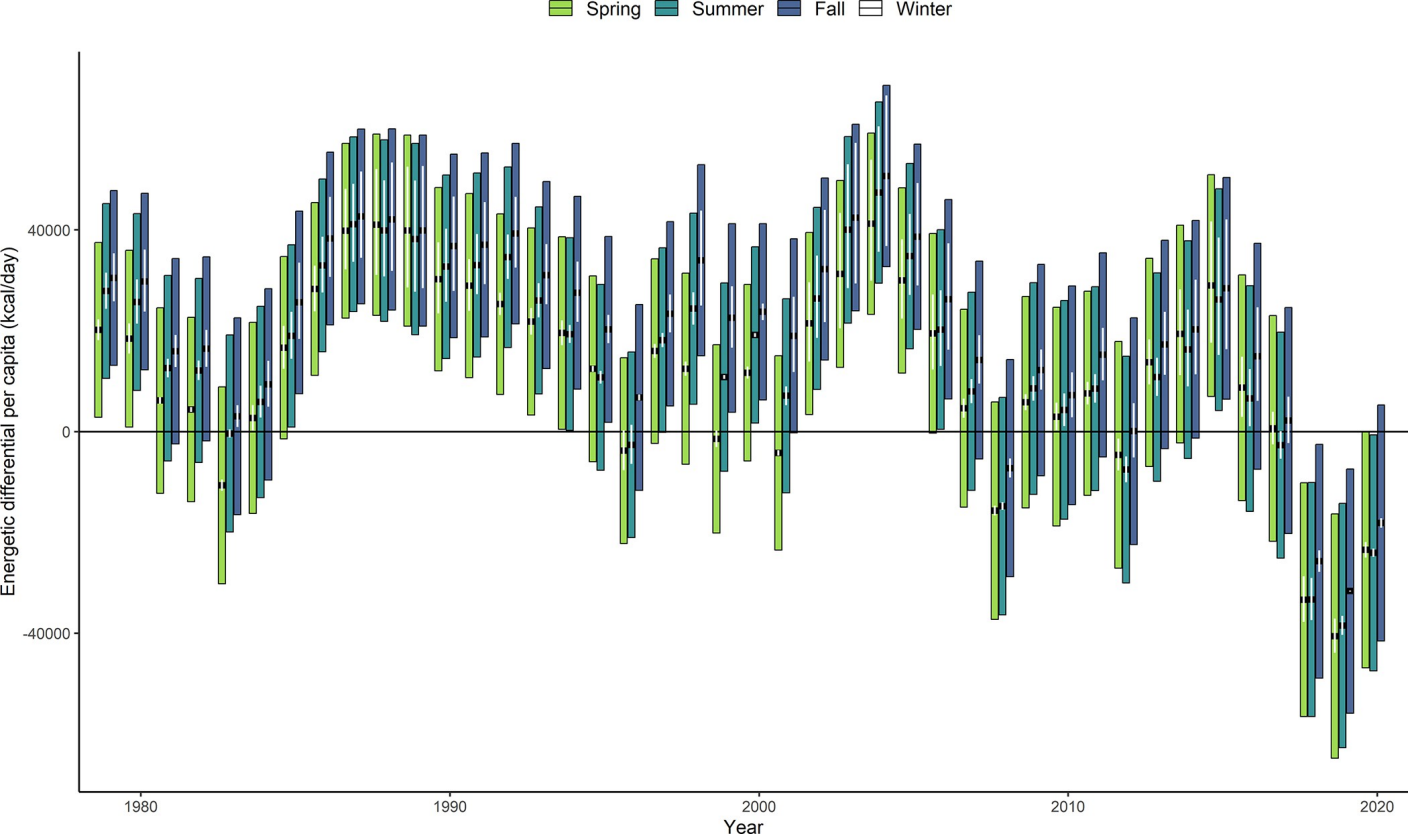
Energetic differential (kcal/day) between the energy ingested and the average DPER per whale individual. Each individual bar represents the highest and lowest DPER bound estimated by Noren et al. (2011), while the thick black dot represents the average DPER differential. The energetic differential was estimated in the spring, summer, and fall between 1979 and 2020. The white vertical lines represent the average DPER differential estimates when running the model with *T*_*s*_ = 5h±20%.

Although SRKW appear to not be limited by prey for most years, our model predicted they were in average energetic deficit in all three seasons for four years between 1979 and 2020 (2008, 2018, 2019, 2020) (Fig 5). When considering high bound DPER and low bound DPER, SRKW were in energetic deficit in all seasons for 19 years and one year, respectively. When combining all three seasons over the last three years of the model, the average energetic differential was -28.716 kcal, which represents about 16.7% of the average adult killer whale DPER. Moreover, while the average number of fish eaten per whale individual decreased by only 3.5% before and after 2017, the average decrease in weight eaten was twice as important (-7.65%). Additionally, the results of our binomial logistic regression suggested that the abundance of age-4 and age-5 Chinook salmon were the most important factors determining whether SRKW met their DPER, with p-values equaling 0.00930 and 0.00086 respectively. Our model also revealed seasonal variation in whether DPER’s were met, with SRKW consistantly consuming less energy in the spring and summer than during the fall. Over the last 40 years, the model predicts that SRKW have been in average energetic deficit during the spring or summer for four additional years. By combining the average energetic differential between the three seasons, we estimated that the SRKW population was in overall energetic defficiency with respect to the stocks and species included in the model for six of the last 40 years (1983, 2008, 2012, 2018, 2019, 2020).

**Fig 6:**
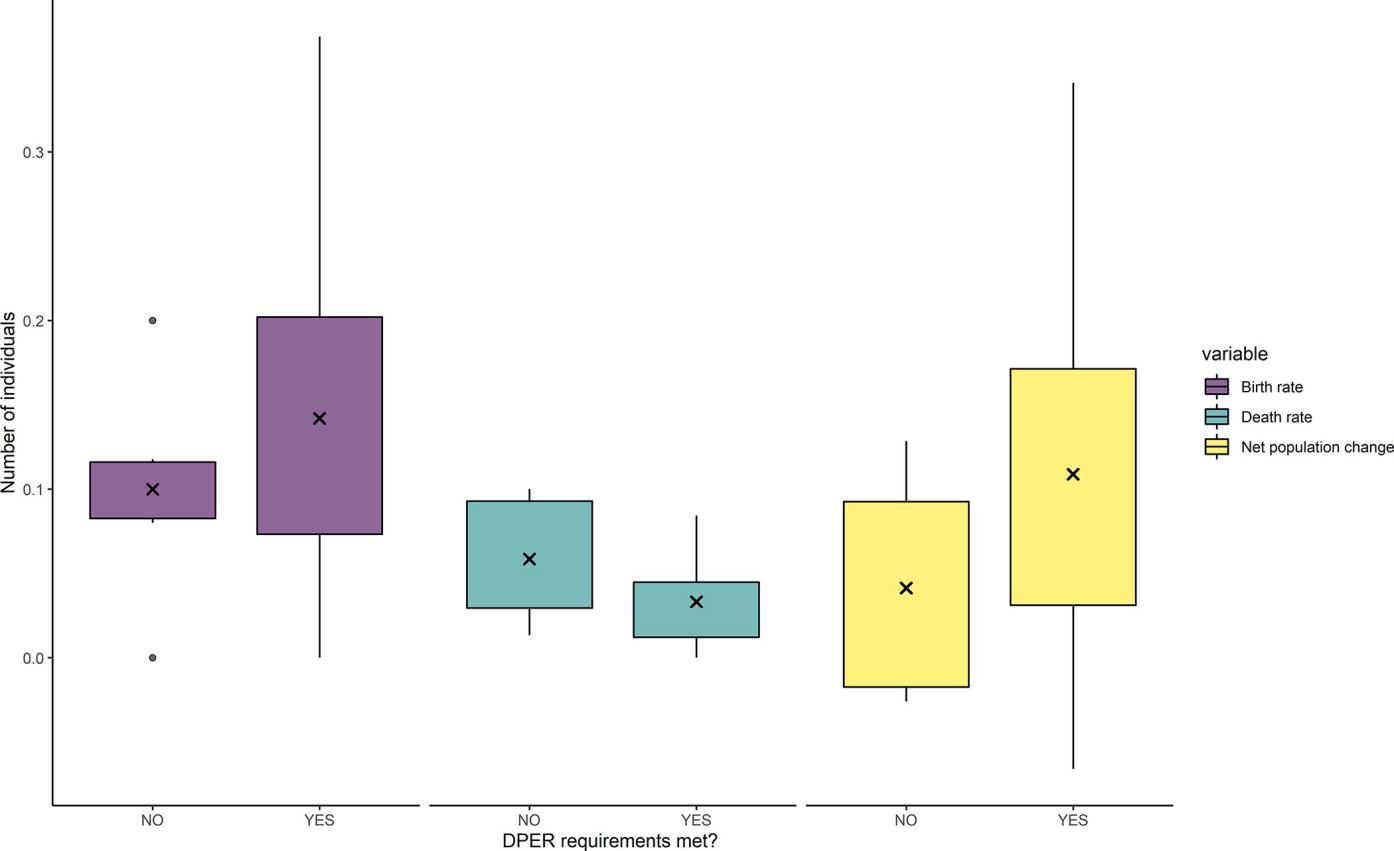
Whisker plot representing the number of births, deaths, and net population growth compared between years where SRKW did not meet their DPER (during at least one season over the year) and years where DPER were met in all seasons. The cross represents the mean.

Our model indicated that the average birth rate and net population change were slightly higher in years during which SRKW met their DPER, while the death rate was slightly higher in years during which SRKW did not meet their DPER (Fig 6). However, no significant results emerged regarding those differences. In all cases, the statistical weight of our results needs to be interpreted with caution with such a small population size. It is also important to note that the overall trend in births, deaths, and annual net population change was similar under all three scenarios.

**Fig 7:**
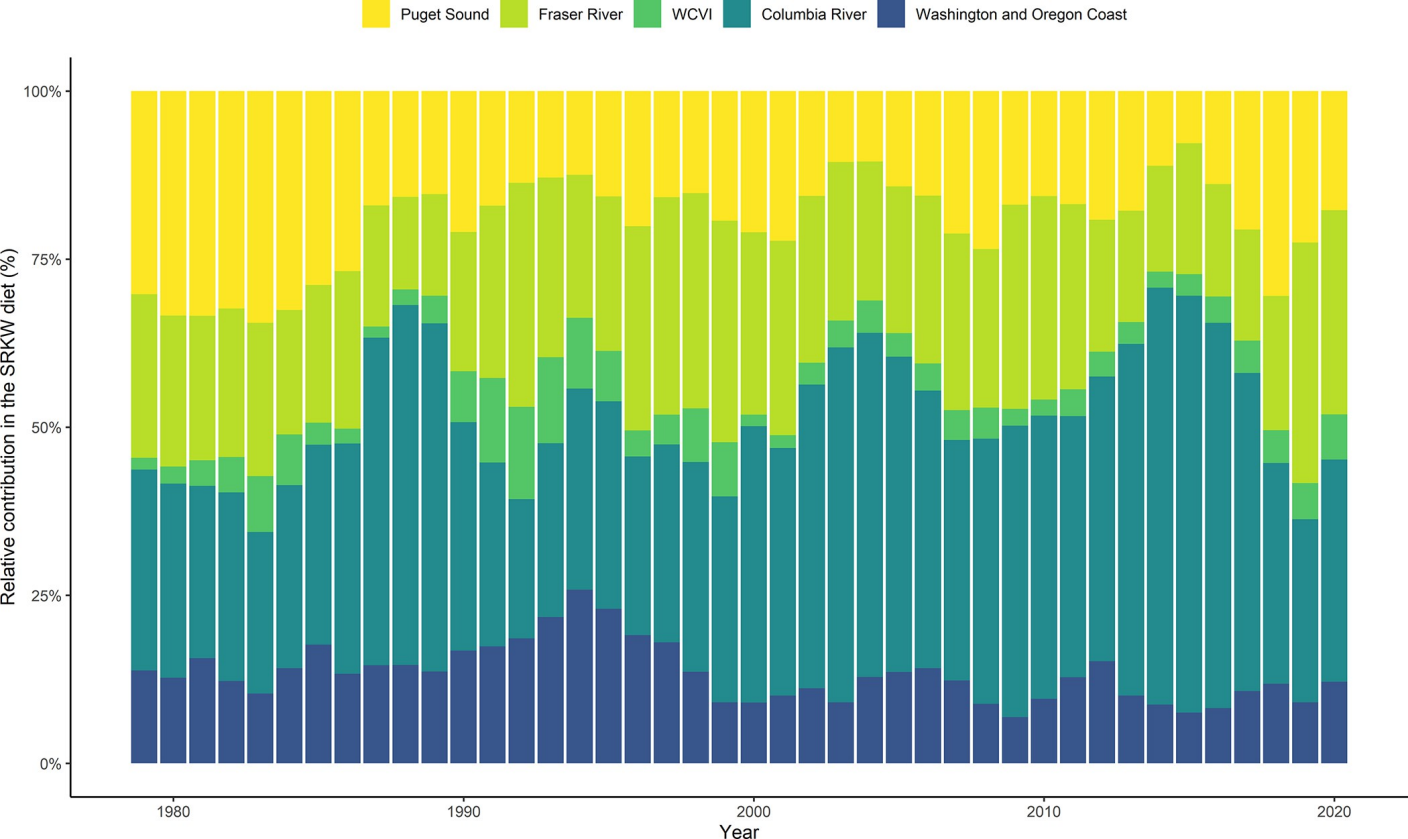
Estimated relative proportion (in %) of different stocks in the predicted SRKW diet from 1979 to 2020. For clarity, the stocks were split into five geographical areas: Puget Sound (NKF, NKS, PSY, PSN, PSF, SNO, SKG, STL), Fraser River (FS2, FS3, FSS, FSO, FCF, FHF, LGS, MGS), WCVI (WVN, WVH), Columbia River (URB, SUM, MCB, CWS, CWF, WSH, LYF, SPR, BON), and Washington Coast and Oregon Coast (WCH, WCN, NOC).

Our model found substantial inter-annual variation in the relative proportion of different Chinook salmon stocks consumed by SRKW (Fig 7). Chinook salmon originating from the Fraser River, the Columbia River, and Puget Sound made up the majority of the predicted SRKW diet from 1979 to 2020, representing an average of 82% over the years. The relative proportion of stocks originating from the Columbia River increased substantially from 1979 (~27% of the SRKW diet) to 2014 (~61% of the SRKW diet). Conversely, the relative proportion of stocks originating from Puget Sound decreased after 1986 (representing an average of ~33% of the diet prior to 1986, against ~18% from 1986 to 2020) (Fig 7).

**Fig 8:**
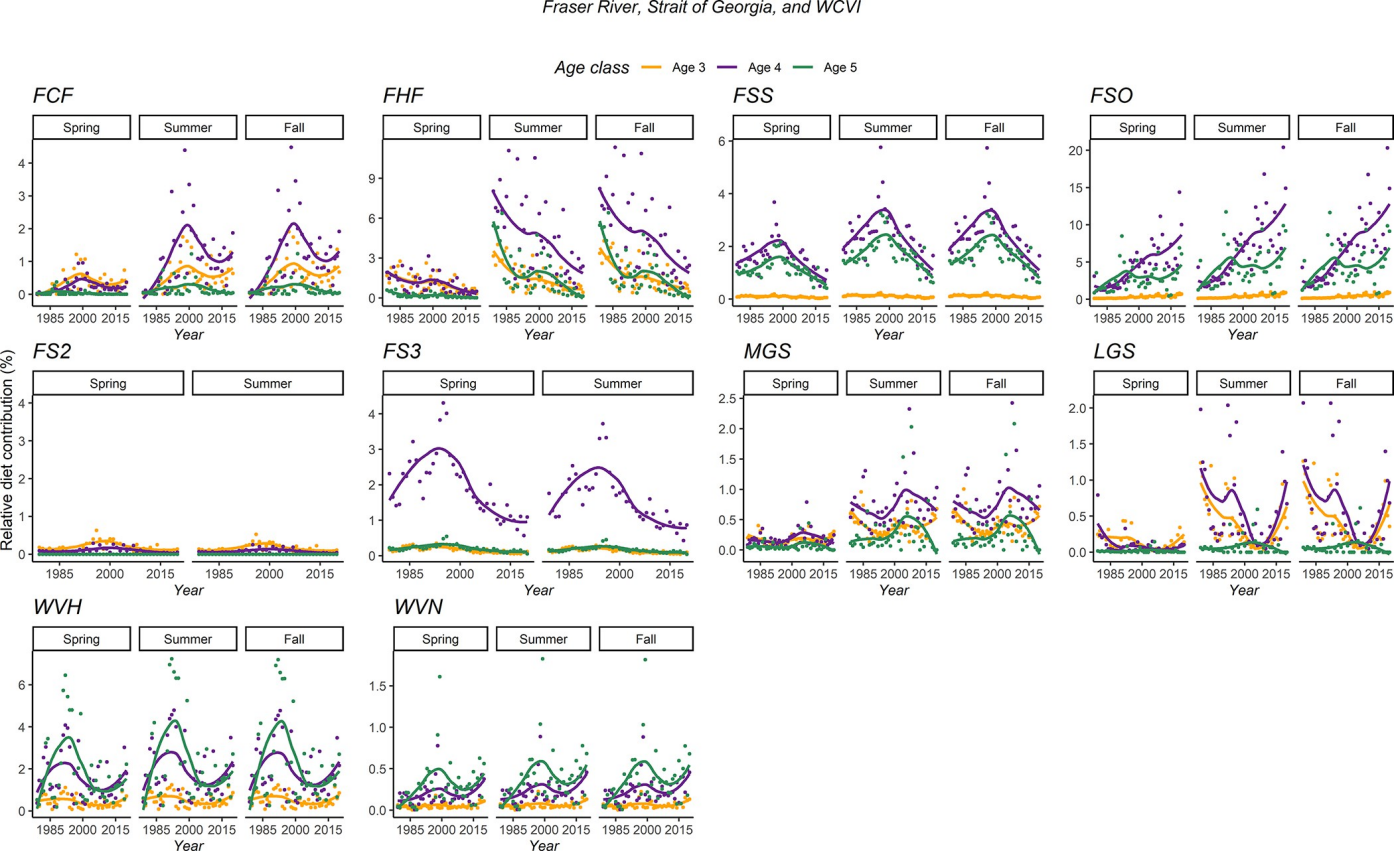
Estimated relative proportion of age-3, age-4, and age-5 Chinook salmon in the SRKW diet from 1979 to 2020. The stock IDs refer to the PSC model, and all stocks are originating from the Fraser River, Strait of Georgia, and West Coast of Vancouver Island.

For the Fraser River, the relative contribution of FCF (age-3 and age-4) and FSO (age-4 and age-5) has increased in all seasons over the last 40 years. For instance, age-4 fish originating from FSO represented less than 4% of the predicted SRKW diet in 1979, against about 18% in 2019. Conversely, the relative proportion of other stocks in the SRKW diet consistently decreased from 1979 to 2020. Age-4 fish originating from FHF declined consistently in the summer and the fall, representing about 7.7% of the SRKW in 1979 against 2.3% in 2020. All stocks originating from the Fraser River or the Strait of Georgia showed a highest contribution in age-4 fish, whereas the highest contribution from the West Coast of Vancouver Island stocks (both hatchery and natural populations) were age-5 Chinook. Finally, most of the stocks originating from the Fraser River, Strait of Georgia, or West Coast of Vancouver Island showed a peak in their relative SRKW diet contribution over some years, mainly around the early 1990s (WVH, FS3) or the early 2000s (FCF, FSS, WVN) (Fig 8).

**Fig 9:**
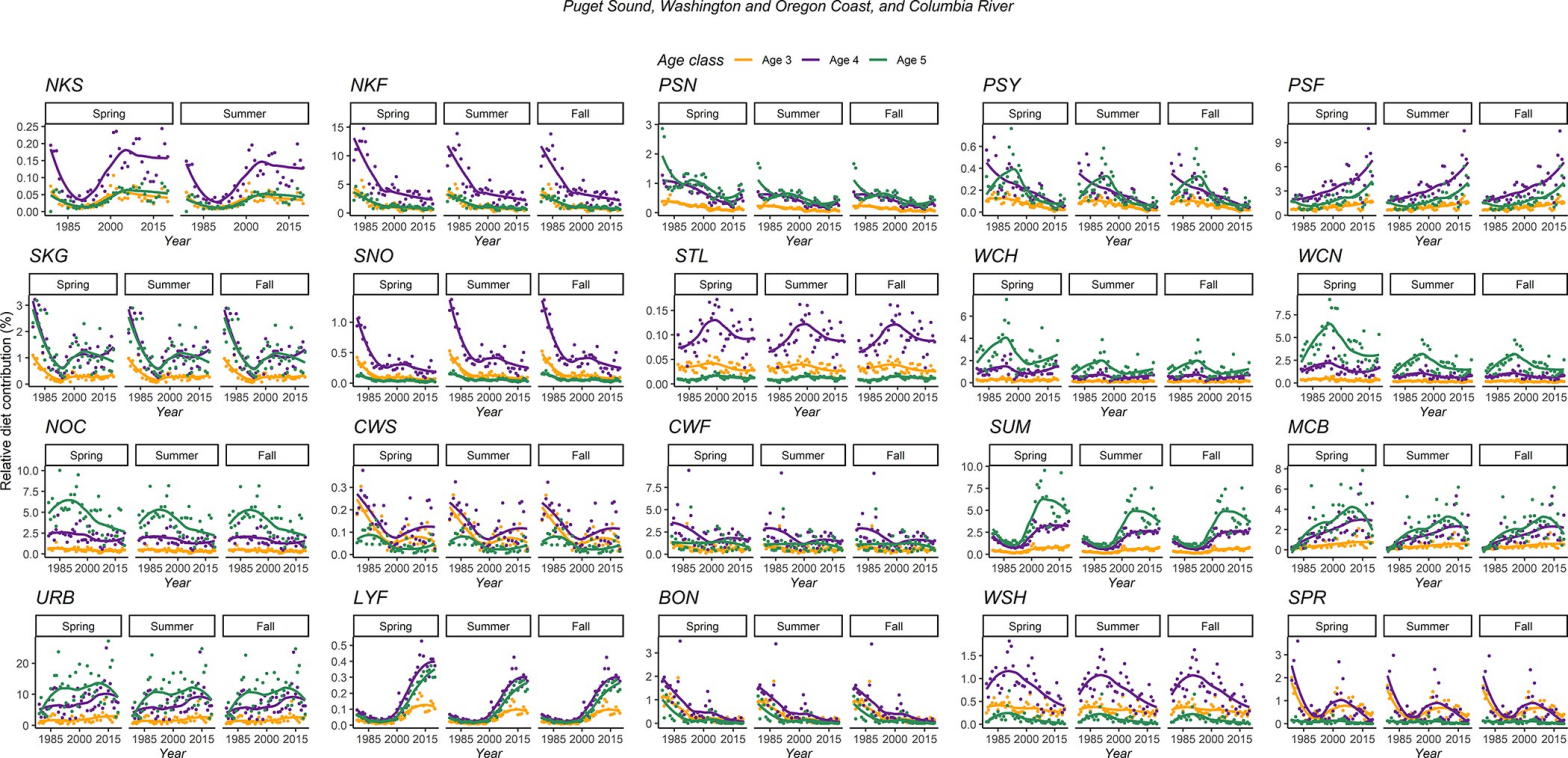
Estimated relative proportion of age-3, age-4, and age-5 Chinook salmon in the SRKW diet from 1979 to 2020. The stock codes refer to the PSC model, and all stocks are originating from Puget Sound, Oregon and Washington Coast, and the Columbia River.

The relative SRKW diet contribution of a majority of stocks originating from Puget Sound decreased substantially between 1979 and 2020. Averaged across all seasons and fish-at-age, a decrease was observed for NKF (-78%), PSN (-71%), PSY (-77.6%), SKG (-62%), and SNO (-76%). Except NKF, all those stocks combined already represented a small portion (<15%, all ages included) of the modeled SRKW diet in 1979. Conversely, the contribution of fish originating from PSF have consistently increased between 1979 and 2020 (+63%). Chinook salmon originating from the Washington and Oregon Coast (NOC, WCH, and WCN) represented in average 12.7% of the SRKW between 1979 and 2020. For the Columbia River, age-5 only Chinook salmon originating from URB represented about 11% of the predicted SRKW diet since 1990 (Fig 9).

## Discussion

Our model predicted that the SRKW population has been in energetic deficit for six of the last 40 years, notably from 2018 to 2020. Several previous studies have highlighted positive correlations between Chinook salmon abundance and SRKW body condition [73], survival rate [39], and fertility [40]. Although our analysis did not permit to support those findings with certainty, our model suggests that SRKW could have a higher birth rate and net population and a lower mortality rate in years during which their DPER was met. Those results suggest that nutritional stress could influence the reproductive ability and survivorship of the whales during years of low prey abundance [19, 40]. Previous studies suggested that such pattern could reflect a trade-off between using energy for other physiological needs than reproduction when resources are scarce [40]. For example, the potential energetic cost associated with swimming further distances to find scattered preys would likely represent an additional metabolic cost and energetic expenditure for SRKW [45]. High death rate also occurred in years during which SRKW met their DPER (1996, 1998, 2001, 2006, 2010, and 2016), but some of those adult individual deaths appeared to be driven by factors unrelated to nutritional stress, such as blunt force trauma (individual ‘J34’), fungal infection (individual ‘L95’), or separation from the rest of the pod (L98) [71]. Over most of the years where SRKW did not meet their DPER, the population has also exhibited a high calf’ mortality rate (at least 50% of the calves dying during their first year of life) and/or a low birth rate. Unfortunately, the low statistical power of our analysis on such a small population did not allow us to identify a significant relationship between energetic differential and those factors.

Our model also predicted that SRKW consistently consumed less calories in the spring than during the fall. This finding supports previous research showing that SRKW body condition is usually at his lowest level before summer, and confirms that SRKW could undergo a more acute nutritional stress during the spring. Moreover, our results support the hypothesis that SRKW might not have enough resources in the spring months in the Salish Sea, therefore spending less time in this critical habitat during this season [74]. Interestingly, our model predicted that SRKW would consume more Chum salmon than any age classes of Chinook salmon during years where their usual Chinook salmon preys were at low levels. This result indicates that SRKW could switch to alternative targets when the abundance of their primary prey decline, which could promote resilience of the population. Although scientists have agreed that SRKW are highly specialized on Chinook salmon, our predictions are in line with recent evidence suggesting that SRKW exhibit seasonal variation in their diet [43]. In this context, future conservation and fisheries management decisions aiming at protecting food resources targeted by SRKW should focus on the recovery of different Pacific salmon, including chum salmon. One major caveat of our model is that e the salmon abundance data used only concern a subset of Chinook stocks, and likely do not include all the fish available to SRKW [53]. For instance, SRKW have been shown to also feed on Chinook stocks originating from the Central Valley or Klamath River (California) during the early Spring [43]. We assume that the Chinook stocks that are not included in this model follow the same population trends as our reference stocks, as they are likely subject to the same natural mortality, fishing pressure, and predation rate. Therefore, we believe that the overall trend in energetic differential for SRKW would remain the same even if those additional stocks were included.

Our model also revealed that age-4 and age-5 Chinook salmon abundances were significant predictors of energetic shortages for SRKW. This finding is not surprising, as older fish would provide more calories to SRKW, which appear to preferably target those Chinook salmon age classes independently of their relative abundance [19]. Age-4 Chinook salmon have been harvested in equivalent or higher numbers than age-3 Chinook salmon since the early 2000s [53]. Over the last 20 years, an annual average of 83,590 age-4 Chinook salmon was harvested by both marine commercial and First Nation marine fisheries in the Salish Sea and along the West Coast of Vancouver Island. According to our model’s predictions, these catch levels are equivalent to the average annual number of age-4 Chinook salmon (~85,900) that SRKW would have consumed over those years. Our model’s predictions thus reinforce the importance of implementing size-specific selectivity for Chinook salmon fisheries operating along the Northeastern Pacific Coast, as well as the use of fishing techniques promoting the survival of larger individuals. Although increasing Chinook salmon abundance for older age classes could promote the recovery of the SRKW population, one important limitation of our model was that we could not account for prey accessibility when considering prey intake rate by SRKW. For instance, previous studies have shown that underwater noise pollution associated with vessel traffic could negatively affect the foraging behavior of resident killer whales, which could further reduce their search rate of efficiency and prey consumption rate [11]. Resident killer whales also travel significantly more in the presence of boats, likely increasing their average DPER [11]. Evaluating how such physical disturbances could influence SRKW foraging abilities is essential to differentiate the relative importance of prey abundance and prey availability on the SRKW prey energetic intake.

Our model predicted that SRKW have consumed in average between 216,000 and 166,000 Chinook salmon annually between 1975 and 2020. This estimate is slightly lower than Chasco et al. (2017), who predicted that SRKW could consume the equivalent to 190,000 and 260,000 Chinook salmon annually in the Salish Sea. This difference is not surprising, as Chasco et al. estimated SRKW Chinook consumption over all seasons, and assumed that SRKW were to meet their DPER. Between 1979 and 2020, our model predicted that SRKW consumed an average of 193,103 Chinook salmon (including all age classes), while an average of 458,669 were harvested by marine fisheries in the Salish Sea and along the West Coast of Vancouver Island over those years. Although Chinook salmon fisheries in marine waters have been drastically reduced since 2000s, the annual average number of adult Chinook salmon harvested by fisheries between 2010 and 2020 was 198,530 while about 187,200 fish were consumed annually by SRKW during this period. Our model thus suggests that SRKW have been consuming slightly less adult Chinook salmon than fisheries have harvested. Although the retention of Chinook salmon is already not permitted for most of those fisheries, our research further highlights the need to implement and reinforce the use of size-selective fishing gear in the area. On the other hand, several recent studies have demonstrated an increasing pinnipeds’ predation pressure on Chinook salmon [32, 35, 36]. For instance, the harbor seal population in the Salish Sea is thought to have been at carrying capacity (~50,000 individuals) since the mid-1990s, and each seal is estimated to consume a daily average of 0.02 adult Chinook salmon [75, 76]. In 2020, our model predicted that SRKW would consume about 800 adult Chinook salmon per day, against 1,000 for harbor seals based on those previous estimates. Our results thus support the need for promoting Chinook salmon population recovery, and highlights the necessity of considering both predation and fisheries pressures on those populations when implementing future conservation goals.

Finally, our model predicted that the overall relative contribution of stocks originating from the Columbia River in the modeled SRKW diet has increased over the last 40 years, while the contribution of stocks originating from Puget Sound has decreased. When presenting those results, we assume to reflect abundance variations of different Chinook salmon stocks available to SRKW, which might partially explain why SRKW have been seen less frequently in the Salish Sea in recent years [74]. Chinook stocks of the Salish Sea have been reduced by about 60% since 1984, and SRKW could logically be feeding upon other more abundant Chinook salmon stocks passing through different areas [77]. This is the case for Columbia River Chinook salmon, which are usually caught off the west coast of Vancouver Island and rarely migrate inside the Salish Sea [55]. Our model also predicted that the SRKW consumption originating from Puget Sound slightly raised around the late 1990s, which is in line with the highest number of sightings of SRKW in the area during those years [51]. Both results support the hypothesis that SRKW occurrence patterns could be driven by food resource abundance and availability. Some stocks—URB, MCB, SUM (Columbia River), FSO (Fraser River), PSF (Puget Sound), WCN and NOC (Washington and Oregon Coast)—showed a high contribution in age-4 and age-5 Chinook salmon in the predicted SRKW diet. As older fish are critical to SRKW meeting their DPER, it appears essential that future Chinook salmon fisheries conservation initiatives direct their efforts towards specific stocks and specific age classes. Altogether, our model provides an overview of prey availability for SRKW, and partially supports the hypothesis that the SRKW population could be limited by food resources. Regarding future research, Furthermore, the use of this model could be useful to evaluate the energy balance of other salmon-eating killer whale population, such as the NRKW. Such future research could help determine whether the differences in prey availability are indeed a plausible cause of the difference in growth trajectories among different resident killer whale populations.

Furthermore, evaluating to which extent external physical disturbances could affect the foraging abilities of SRKW is critical to identify whether SRKW could still fail to meet their daily prey energetic requirements under more favorable prey abundance scenarios. Finally, it will be key to understand how changes in fisheries harvest practices and marine mammals’ predation pressure could influence the abundance of prey available to SRKW.
